# Editorial: Resilience in Chronic Disease

**DOI:** 10.3389/fpsyt.2022.846370

**Published:** 2022-02-14

**Authors:** Zeng Jie Ye

**Affiliations:** School of Nursing, Guangzhou University of Chinese Medicine, Guangzhou, China

**Keywords:** resilience, chronic disease, vulnerability, cognition, intervention, psychosomatic

Resilience is usually defined as one's ability to “bounce back” from adversity and is a salient indicator of the quality of life and psychosocial functions of patients facing chronic diseases, for instance, cancer, hypertension, irritable bowel syndrome, and heart failure, etc. (1, 2). Thus, resilience is an important attribute for patients who face the challenge of chronic disease. How patients gain or lose resilience resources during the diagnosis, treatment, and ultimately the survival of chronic disease is attracting increased attention in bio-psycho-social medicine. Interestingly, there exist many different concepts of resilience including physiological, psychological, social, and spiritual resilience, that involve a variety of factors, from behavioral constructs like defense mechanisms, beliefs, and personalities to molecular levels of brain-derived neurotrophic factor, neuropeptide Y, and oxytocin in emotion- and cognition-related brain areas (3, 4). The factors underpinning psychological and social resilience are less established and therefore the primary focus in this collection. However, the construct of resilience has not been established and whether resilience should be defined as a state or trait continues to be debated (5). This collection aims to fill gaps in knowledge regarding the predicted ability of resilience to enhance long-term quality of life and other psychosomatic outcomes in patients with different chronic diseases, which need to be further explored and clarified.

The call for submissions on *Resilience in Chronic Disease* received a great response. The collection consists of 12 studies with a total of over 7,100 participants from different countries. A diversified array of populations including the general population in the COVID-19 pandemic (Büssing et al.), patients with chronic pain (Orakpo et al.), cancer (Tang et al.), irritable bowel disease (Funaba et al.; Luo et al.), cardiovascular disease (Qiu R. et al.), neurocognitive disorders (Wang et al.), renal transplant (Hu et al.), and rheumatoid arthritis (Shen et al.), as well as caregivers for children with chronic illness (Qiu, Xu et al.), patients with liver cancer (Mao et al.), and maintenance hemodialysis (Qiu, Huang et al.), were examined. Findings derived from the current collection echo the existing positive-psychology literature emphasizing the importance of psychological and social resilience in the move forward with chronic disease. Scientists from multi disciplines have contributed to this collection, providing contributions that raise awareness, educate, and reduce the impact of different chronic diseases on patients and their caregivers. However, several limitations should be emphasized here. First, most articles used observational designs (i.e., Büssing et al.; Tang et al.; Luo et al.; Qiu, Huang et al., etc.), describing resilience and its associations with other aspects of the psychological, social, and physical well-being of patients with chronic disease. Our specific aim was first, to recognize distinct resilience trajectories during diagnosis, treatment, and survivorship throughout the course of chronic disease, which was not achieved in the current collection (6, 7). Second, articles about resilience theory and instrument development are limited, and more future research should be undertaken to develop new resilience theories and instruments for the cultural and developmental levels of measuring the psychological and social aspects of resilience, which can help address debates about resilience construction (8–10). Third, the predicted ability of resilience to enhance the long-term quality of life and other psycho-somatic outcomes in patients with different chronic diseases is not fully explained (11). Prevention-oriented studies investigating how resilience mitigates the effect of chronic disease on patients' health in different phases (i.e., first diagnosis, remission, relapse) should be further explored. Fourth, in the move forward with resilience research in chronic disease, investigating the efficacy, sustainability and implementation challenges of resilience programs targeting patients with chronic disease and their caregivers should be the next step in furthering knowledge [Seiler et al.; (12–15)].

## Author Contributions

The author confirms being the sole contributor of this work and has approved it for publication.

## Funding

This research was funded by grants from the National Natural Science Foundation of China (No. 71904033), the Humanity and Social Science Youth Foundation of the Ministry of Education of China (No. 19YJCZH227), the Humanity and Social Science Foundation of Department of Education of Guangdong Province (No. 2020WTSCX009), the Humanity and Social Science Foundation of Guangzhou (No. 2021GZGJ57), and the Humanity and Social Science Foundation of Guangzhou University of Chinese Medicine (Nos. 2020SKXK01 and 2021SKYB07).

## Conflict of Interest

The author declares that the research was conducted in the absence of any commercial or financial relationships that could be construed as a potential conflict of interest.

## Publisher's Note

All claims expressed in this article are solely those of the authors and do not necessarily represent those of their affiliated organizations, or those of the publisher, the editors and the reviewers. Any product that may be evaluated in this article, or claim that may be made by its manufacturer, is not guaranteed or endorsed by the publisher.
